# Photoperiod Modulates Fast Delayed Rectifier Potassium Currents in the Mammalian Circadian Clock

**DOI:** 10.1177/1759091416670778

**Published:** 2016-10-03

**Authors:** Sahar Farajnia, Johanna H. Meijer, Stephan Michel

**Affiliations:** 1 Leiden University Medical Center, Leiden, The Netherlands; 2 Netherlands Institute for Neuroscience, Amsterdam, The Netherlands

**Keywords:** circadian rhythms, fast delayed rectifier current, photoperiod, potassium channels, seasonality, suprachiasmatic nucleus

## Abstract

One feature of the mammalian circadian clock, situated in the suprachiasmatic nucleus (SCN), is its ability to measure day length and thereby contribute to the seasonal adaptation of physiology and behavior. The timing signal from the SCN, namely the 24 hr pattern of electrical activity, is adjusted according to the photoperiod being broader in long days and narrower in short days. Vasoactive intestinal peptide and gamma-aminobutyric acid play a crucial role in intercellular communication within the SCN and contribute to the seasonal changes in phase distribution. However, little is known about the underlying ionic mechanisms of synchronization. The present study was aimed to identify cellular mechanisms involved in seasonal encoding by the SCN. Mice were adapted to long-day (light–dark 16:8) and short-day (light–dark 8:16) photoperiods and membrane properties as well as K^+^ currents activity of SCN neurons were measured using patch-clamp recordings in acute slices. Remarkably, we found evidence for a photoperiodic effect on the fast delayed rectifier K^+^ current, that is, the circadian modulation of this ion channel’s activation reversed in long days resulting in 50% higher peak values during the night compared with the unaltered day values. Consistent with fast delayed rectifier enhancement, duration of action potentials during the night was shortened and afterhyperpolarization potentials increased in amplitude and duration. The slow delayed rectifier, transient K^+^ currents, and membrane excitability were not affected by photoperiod. We conclude that photoperiod can change intrinsic ion channel properties of the SCN neurons, which may influence cellular communication and contribute to photoperiodic phase adjustment.

## Introduction

Organisms have developed an endogenous circadian clock to adapt their physiology and behavior with daily and seasonal changes. In mammals, the suprachiasmatic nucleus (SCN) of the anterior hypothalamus serves as a central circadian clock controlling rhythms in other brain areas and in peripheral tissues (Silver and Kriegsfeld, 2014). Most SCN neurons show a sinusoidal pattern in electrical activity rhythm with a peak in action potential frequency during the middle of the day. Behavioral activity is triggered and arrested at the half-maximum level of this sinusoidal SCN rhythm (Houben et al., 2014). Besides its role as a daily timekeeper, the SCN is able to encode the seasonal change in day length by adjusting the composite pattern of electrical activity (Mrugala et al., 2000; Vanderleest et al., 2007; Brown and Piggins, 2009).

Seasonal changes in day length modulate the waveform of the SCN electrical activity rhythm resulting in a broad peak during long summer days and a narrow peak in short winter days (Sumova et al., 1995; Schaap et al., 2003; Vanderleest et al., 2007; Brown and Piggins, 2009). The electrical activity is an important output of the SCN which affects behavioral activity patterns. In accordance to the altered waveform in electrical activity, mice shorten the duration of their behavioral activity in long photoperiods and lengthen the duration in short photoperiods.

The seasonal change in SCN waveform is based on a phase redistribution of individual neuronal activity rhythms. In short days, the cells are more synchronized in phase, while in long days they become desynchronized. This is the case both for SCN single cell electrical activity patterns and for molecular expression profiles (Vanderleest et al., 2007; Naito et al., 2008; Brown and Piggins, 2009). Altered phase distribution of clock genes expression such as *Period 1*, *Period 2*, *Rev-erb*, and *Dbp* was observed in long-day and short-day photoperiod (Hazlerigg et al., 2005; Inagaki et al., 2007). At the single cell level, neither the profile of clock gene expression (Naito et al., 2008) nor electrical activity patterns (Vanderleest et al., 2007; Brown and Piggins, 2009) were influenced by photoperiod. Therefore, the plasticity within the SCN network accounts for the change in the ensemble waveform of neuronal activity and of clock gene expression to a narrower phase distribution in short (8 hr) winter days compared with a broader phase distribution in long (16 hr) summer days (Rohling et al., 2006; Vanderleest et al., 2007; Brown and Piggins, 2009). There is evidence for a role of intercellular communication within the SCN in photoperiodic phase adjustment, mainly via neurotransmitters like vasoactive intestinal peptide (VIP; Lucassen et al., 2012) and gamma-aminobutyric acid (GABA; Evans et al., 2013; Myung et al., 2015).

The seasonal adaptation of network properties seems to be based on changes in neurotransmission and receptor function. In the SCN, photoperiodic cues can switch the GABAergic function from inhibitory to excitatory by changing the equilibrium potential of GABA (Farajnia et al., 2014; DeWoskin et al., 2015; Myung et al., 2015). In paraventricular and periventricular nuclei of rats exposed to different photoperiods, a switch between somatostatin and dopamine neurotransmitters has been reported via modification in the number of intracellular storage vesicles as well as changes in postsynaptic receptor population (Dulcis et al., 2013). These findings indicate that cellular changes occur in paraventricular, periventricular, and SCN nuclei of hypothalamus under the influence of photoperiod. Despite considerable knowledge of the neurotransmitters and neuropeptides involved in intercellular communication within the SCN, the mechanisms of synchronization and photoperiod-induced phase dispersal are still inconclusive. It is evidenced that intrinsic neuronal properties of the SCN are modulated in a circadian fashion under the control of different kinds of ionic currents (Colwell, 2011). Potential changes in the neuronal properties and ionic channels activity—affecting excitability and therefore the degree of synaptic interaction—have not been investigated under different photoperiods and are the focus of the present study.

We performed patch clamp experiments in acutely prepared slices of the SCN from mice which had been entrained to either long-day (16 hr) or short-day (8 hr) photoperiod. We measured the activity of three different K^+^ currents—fast delayed rectifier (FDR), A-type current (I_A_), and slow delayed rectifier (SDR)—as well as spike frequency and passive membrane properties during the day and at night. We also evaluated the action potential (AP) waveform in different photoperiods. We report an altered circadian modulation of FDR current activity induced by long-day photoperiod with higher amplitude at night compared with short-day photoperiod while no photoperiod-induced differences were observed in the frequency of action potentials and the amplitude of I_A_ and SDR currents. The enhanced FDR current did shorten action potential duration and increased afterhyperpolarization (AHP), which is discussed to modify efficiency of synaptic communication.

## Material and Methods

### Animals and Housing

Male C57BL/6 mice (Harlan, Horst, The Netherlands, 100 days old; *n* = 41) were individually housed in cages equipped with a running wheel and ad libitum access to food and water. Up to nine cages were placed in light-tight cabinets under constant temperature and adjustable light regimes. Animals were adjusted to the long-day (16 hr light or 8 hr dark) or short-day (8 hr light or 16 hr dark) photoperiod for a minimal period of 4 weeks to warrant the full adaptation of the SCN clock to the given photoperiod (Vanderleest et al., 2007). All experimental procedures were approved by the Committee on Animal Health and Care of the Dutch government (no. 11010).

### Slice Preparation

On the experimental day, animals were anesthetized (2% isofluorane) and killed for slice preparation as described previously (Farajnia et al., 2012). In brief, brain slices containing the SCN were transferred to a recording chamber (RC-26 G; Warner Instruments; Hamden, CT) and placed on the stage of an upright microscope (Axioskop FS-2, Zeiss; Oberkochen, Germany) for the patch clamp experiments. Slices were continuously perfused with artificial cerebrospinal fluid (pH 7.2 −7.4), containing (in mM): 116.4 NaCl, 5.4 KCl, 1 NaH_2_PO_4_, 0.8 MgSO_4_, 1.8 CaCl_2_, 23.8 NaHCO_3_ and 16.7 D-glucose (Sigma-Aldrich). Recordings were performed at external time 12 ± 3 hr and external time 0 ± 3 hr. External Time 12 is defined as the middle of the day in a given photoperiod (Daan and Merrow, 2002).

### Whole Cell Patch Clamp Recordings

Micropipettes of 5 to 7 MΩ were pulled from borosilicate tubing (WPI B150F-4) with a commercial puller (PC-10 Narishige; London, UK) and filled with an internal solution (pH: 7.2–7.3; osmolality: 290–300 mOsm) containing (in mM) 112.5 K-gluconate, 1 EGTA, 10 Na^+^-HEPES, 5 MgATP, 1 GTP, 0.1 leupeptin, 10 phosphocreatine, 4 NaCl, 17.5 KCl, 0.5 CaCl_2_, and 1 MgCl_2_ (Sigma-Aldrich).

All recordings were performed using a commercial patch amplifier (EPC 10–2; HEKA, Lambrecht/Pfalz, Germany). Action potential frequencies and resting membrane potential were measured in current clamp configuration. Membrane resistance was obtained from the value provided by the amplifier software which uses holding current, holding voltage, and access resistance for its calculation. Thereafter, fast and slow delayed rectifier (FDR and SDR, respectively) and I_A_ potassium currents were measured in voltage clamp configuration. FDR, SDR, and I_A_ currents were isolated as described previously (Farajnia et al., 2012). Briefly, Na^+^ and Ca^2+^ currents as well as spontaneous GABAergic currents were eliminated by application of tetrodotoxin (0.5 μM; Tocris bioscience), cadmium (25 μM), and bicuculine (20 μM; Sigma-Aldrich), respectively. Afterward, tetraethylammonium-Cl^−^ (TEA) in low and high concentrations (1 and 20 mM; Sigma-Aldrich) was applied to block FDR and SDR currents, respectively. Current traces were evoked by 400 ms progressively depolarizing voltage pulses (−50 to +60 mV, 10 mV increments), following a prepulse at −100 mV for 100 ms. FDR currents were isolated by subtracting the current traces acquired in the presence of TEA (1 mM) from the ones obtained in control solution. In addition, digital subtraction of traces in 20 mM TEA from traces obtained in 1 mM TEA (blocking FDR) resulted in isolation of SDR. To characterize I_A_ currents, identical voltage protocol (−60 to +60 mV, 150 ms, 10 mV increments) was applied but with two different prepulses to activate (−90 mV, 150 ms) or inactivate (−45 mV, 150 ms) the current. I_A_ current was isolated by subtracting the traces lacking the I_A_ current from traces containing the current. Peak current amplitude was determined in steady state at the end of the test pulse for SDR and FDR currents and the maximum value of the trace was used as the peak value for the transient I_A_ current. The kinetics of the currents was not analyzed.

### Perforated Patch Clamp Recordings

To evaluate action potential waveform, amphotericin perforated-patch technique was performed in the current-clamp mode as described previously (Farajnia et al., 2015). In brief, micropipettes (6–8 MΩ) were filled with a solution containing (in mM) 112.5 K-gluconate, 4 NaCl, 17.5 KCl, 1 CaCl_2_, 1 MgCl_2_, and 10 HEPES (pH 7.2; 290–300 mOsm). Amphotericin B (240 µg/ml) was prepared freshly and added to the internal solution. The tip of the glass pipette was filled with amphotericin B-free solution to aid the formation of a gigaohm seal. Access resistance stabilized to values between 40 MΩ and100 MΩ within 5 to 10 min after obtaining the seal. While this range of access resistance was not considered for voltage clamp experiments, it is sufficient for obtaining information on the waveform of action potentials in current clamp mode. The access resistance was monitored throughout the experiment to ensure a stable value and detect accidental breakthrough or resealing.

To estimate the contribution of increased FDR current to the AP waveform during the night in long photoperiod, the width of the AP and the amplitude and duration of the AHP were measured. The duration of AP was calculated at 50% of the AP peak. The difference between the resting membrane potential and membrane potential at the trough of the AHP was considered as AHP amplitude. Resting membrane potential was defined as membrane potential 40 ms prior to the AP peak. Duration of AHP was calculated by dividing the AHP area (mV·ms) by the AHP amplitude (mV).

### Data Analysis

In whole cell patch clamp experiment, only recordings with access resistance lower than 40 MΩ were considered in the final analysis. Fit master (version 2.67; HEKA, Lambrecht/Pfalz, Germany), Igor Pro (version 6.22A; Wavemetrics, Portland, OR), MiniAnalysis (version 6.0.7; Synaptosoft, Fort Lee, NJ), and SPSS (version 17.0; IBM, Armonk, NY) were used for statistical analysis. All values were tested first for normality of the data and homogeneity of the variances using Shapiro-Wilk and Levene’s tests, respectively. Unpaired *t* tests in SPSS were applied to peak current values of I_A_, FDR, and SDR to evaluate significant differences (*p* ≤ .05).

## Results

To investigate the impact of photoperiod on the cellular properties of the SCN neuron, passive and active membrane properties—such as voltage dependent K^+^ currents, firing rate, and action potential waveform—were measured in SCN neurons of mice adapted to long-day and short-day photoperiod.

### SDR and I_A_ K^+^ Currents Are Indistinguishable in Long and Short Photoperiod

**Figure 1.**
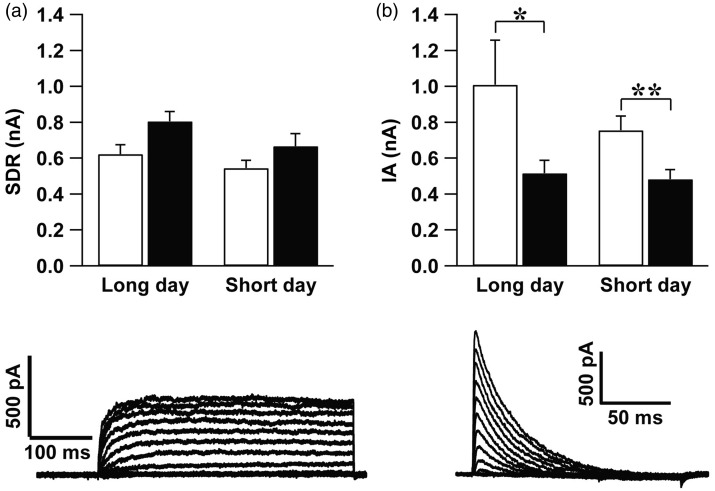
Slow delayed rectifier (SDR) and A-type (IA) K^+^ currents recorded from the SCN neurons did not differ between long-day and short-day photoperiod. (a) Top: The amplitude of the maximum SDR current elicited by a voltage step to +60 mV (mean ± SEM) was not significantly different between day (*n* = 11 cells from four animals) and night (*n* = 5 cells from three animals) in long (*p* = .072) and short (*p* = .156) photoperiod. No differences were found between long-day and short-day photoperiod. Bottom: one typical example of SDR current traces elicited by a series of voltage steps. (b) Top: IA current showed a larger amplitude (mean ± SEM), during the day as compared with the night in long photoperiod. In short photoperiod, the amplitude of this current was also higher during the day than the night. No differences in amplitude of IA current were found between the two photoperiods neither during the day nor during the night. Bottom: an example of IA current traces elicited by a series of voltage steps. *p < .05, **p < .01. (from left to right, (a): 11 cells from four animals; 5 cells from three animal, 7 cells from five animals, 14 cells from six animals; (b):10 cells from six animals, 21 cells from four animals, 20 cells from four animals, 17 cells from three animals).

SDR current does not show a daily modulation under (light–dark) LD 12:12 conditions (Itri et al., 2005). Recordings in long-day (*p* = .072) and short-day (*p* = .156) photoperiod did also not reveal a circadian rhythm in the SDR current amplitude (Figure 1(a)). No difference in the current amplitude was found between short and long photoperiod neither during the day (*p* = .313) nor during the night (*p = *.142, independent student *t* test).

Consistent with previous findings in LD 12:12 cycle (Itri et al., 2010), I_A_ current amplitude was higher during the day as compared with the night both in long-day (*p* = .037) and short-day photoperiod (*p* = .010). No significant differences were found between the animals adjusted to long and short photoperiod (day: *p* = .962, night: *p* = .826, Mann-Whitney test; Figure 1(b)).

In summary, the data show that SDR and I_A_ currents are not affected by photoperiod.

### FDR K^+^ Current Is Enhanced During the Night in Long Photoperiod

**Figure 2.**
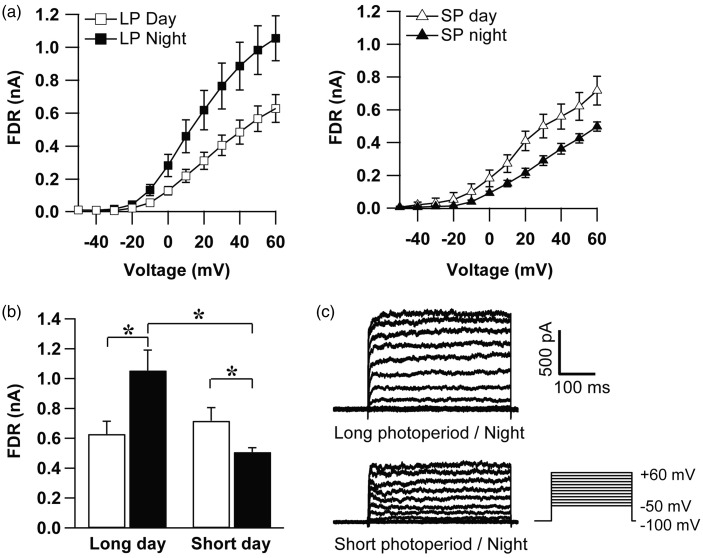
Fast delayed rectifier K^+^ current (FDR) in the SCN neurons was increased at night in long-day photoperiod. (a) Current-voltage relationship of FDR current amplitude (mean ± SEM) revealed a daily rhythm in both long-day and short-day photoperiod. However, in short-day photoperiod (SP) the amplitude of the current was larger during the day, while in long-day photoperiod (LP), this rhythm was reversed. (b) Maximum FDR current amplitude in response to voltage steps to +60 mV (mean ± SEM). During the night (filled bars), the FDR current was significantly increased in long photoperiod compared with short photoperiod. During the day (open bars), the magnitude of FDR currents did not differ between long and short photoperiod (*p* = .8). (From left to right: 12 cells from 4 animals, 6 cells from 4 animals, 8 cells from 6 animals, 18 cells from 10 animals; *p < .05) (c) Examples of FDR current traces elicited by a series of voltage steps (see inset at right) in long and short photoperiod during the night.

In LD 12:12 cycle, FDR current is under a circadian rhythm with a higher magnitude during the day as compared with the night (Itri et al., 2005). A similar rhythm in the magnitude of FDR current was found in short-day photoperiod with significantly higher values recorded in the day compared with the night (*p* = .047; Figure 2(a)right). In long photoperiod, however, the magnitude of the FDR current was higher in the night than in the day (*p* = .0134; Figure 2(a)left). Therefore, the main difference between long-day and short-day photoperiod was recorded during the night when a larger FDR current was observed in long photoperiod (Figure 2(b) and (c); *p* = .0132). During the day, no differences in the FDR current magnitude were distinguished between long-day and short-day photoperiod (*p* = .8, independent student *t* test).

The data show a profound effect of long-day photoperiod on the circadian rhythm in FDR activation.

### Firing Rate, Resting Membrane Potential, and Membrane Resistance Are Not Affected by Photoperiod

**Figure 3.**
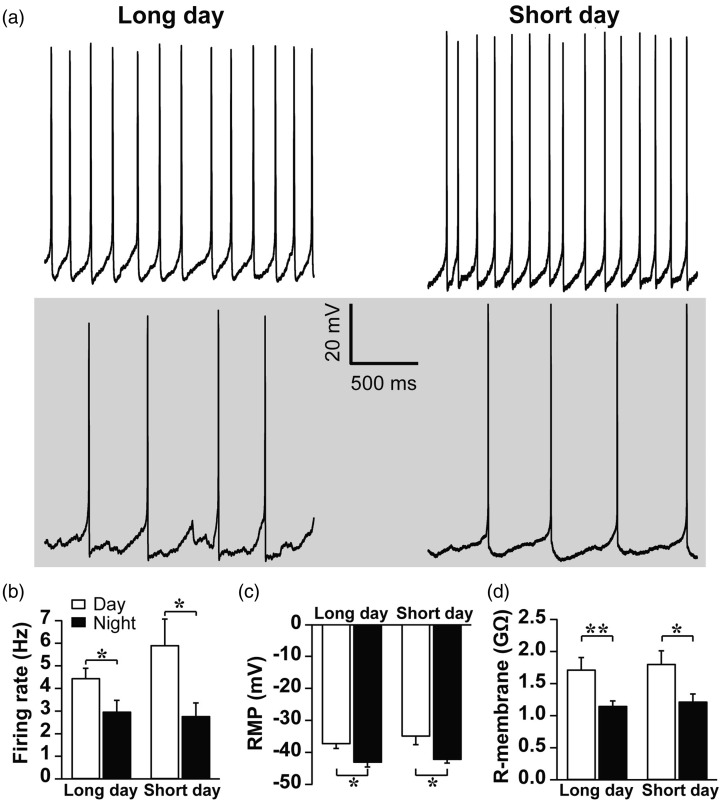
Neuronal electrical activity and passive membrane properties of SCN neurons remained unchanged in long-day and short-day photoperiod. (a) Examples of neuronal activity recorded in current clamp mode. The gray background indicates the night-time recordings. (b) In long-day photoperiod, firing rate (mean ± SEM) was higher during the day than the night which was comparable to short-day photoperiod (from left to right: 30 cells from nine animals, 23 cells from eight animals, 11 cells from eight animals, 17 cells from eight animals; * *p* < .05). (c) Resting membrane potential (RMP; mean ± SEM) was rhythmically modulated in both long-day and short-day photoperiod (*p* < .05). No difference was found between long and short photoperiod neither during the day (*p* = .401) nor during the night (*p* = .633). From left to right: 38 cells from 10 animals, 16 cells from 6 animals, 14 cells from 7 animals 11 cells from 6 animals; *p < .05 (d) Membrane resistance (R-membrane; mean ± SEM) showed a daily rhythm both in long-day and short-day photoperiod. There was no difference between the short and long photoperiod (day: *p* = .774, night: *p* = .631). From left to right: 71 cells from 11 animals, 59 cells from 8 animals, 35 cells from 11 animals, 59 cells from 8 animals; *p < .05, **p < .01.

FDR current is known to be one of the regulators of firing frequency in the SCN (Itri et al., 2005). Our experiments revealed that FDR current increases at night in long photoperiod. Therefore, we measured the frequency of spontaneously generated action potentials of the SCN neurons in different photoperiods. Surprisingly, the firing frequency was comparable in long-day and short-day photoperiod both during the day (*p* = .257) and night (*p* = .797; Figure 3(a) and (b)). Firing rate was significantly decreased at night as compared with the day in cells recorded from animals adjusted to long-day (*p* = .03) and short-day photoperiod (*p* = .013, independent student *t* test).

Resting membrane potential exhibited a circadian rhythm in both long-day (*p* = .017) and short-day photoperiod (*p* = .018, independent student *t* test) with no difference in the amplitude between the photoperiods (Figure 3(c)). Likewise, membrane resistance was rhythmically controlled in both long days (*p* = .007) and short days (*p* = .016, independent student *t* test), and values did not differ between photoperiods (Figure 3(d)).

The data suggest that photoperiod does not affect membrane properties other than FDR activation at night. However, photoperiodic-induced changes in FDR current at night do not modify the firing rate of SCN neurons.

### AP Waveform Is Altered During the Night in Long Photoperiod

**Figure 4.**
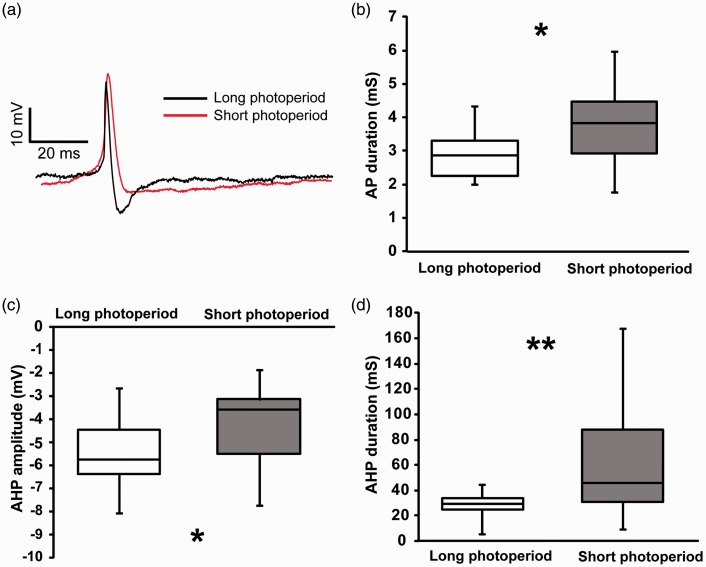
Action potential (AP) waveform of the SCN neurons was altered in long photoperiod. (a) Example traces of APs recorded during the night in long photoperiod (black) and short photoperiod (red). (b to d) Box plot (median and quartile) presentation of AP duration (b), afterhyperpolarization (AHP) amplitude (c), and duration (d) under long (*n* = 15 cells from three animals) and short (*n* = 18 cells from three animals) photoperiods. Stars indicate the significant difference between the mean values (*p ≤ .05, **p < .01).

Since photoperiodic-induced FDR enhancement did not affect firing rate, we investigated if the altered FDR at night would still affect the shape of the AP. To this end, we performed perforated patch recordings in SCN neurons of mice entrained to long and short photoperiod and analyzed the waveform of the APs generated during the night. We determined the width of the AP and the duration and amplitude of the AHP (Figure 4). The duration of action potential was reduced by 25% in long-day versus short-day photoperiod (*p* = .019; Figure 4(a) and (b)). Interestingly, AHP amplitude was increased (*p* = .049) and its duration shortened (*p* = .004) in long-day photoperiod as compared with short photoperiod (Figure 4(a) and (c) to (d)).

The analysis of action potential waveform show an effect of the photoperiod-altered FDR current on spike duration and AHP, which may lead to altered synaptic transmission and cellular communications even though spike frequency is not affected.

## Discussion

To investigate neuronal mechanisms underlying seasonal encoding within the circadian clock, patch clamp recordings were performed in SCN slices from mice entrained to long and short photoperiods. Whole cell recordings showed that the frequency of spontaneous action potentials is not significantly different in animals adapted to either long days or short days. This result is consistent with previous recordings using extracellular electrophysiological recordings (Vanderleest et al., 2007; Brown and Piggins, 2009). Moreover, we showed that membrane resistance and resting membrane potential were also comparable in neurons entrained to long-day and short-day photoperiod.

In this study, we provide the first demonstration that an ionic current is regulated by photoperiod. We observed an upregulation of the amplitude of FDR current during the night in long-day photoperiod, while SDR and I_A_ currents remained unchanged. Interestingly, the amplitude of the FDR current is even higher during the night than during the day, which indicates a reversal of the circadian modulation as compared with recordings from 12:12 LD conditions (Itri et al., 2005; Farajnia et al., 2012) and as compared with recordings from animals adapted to short photoperiod (Figure 2). As a consequence of the altered FDR current magnitude, we observed significant changes in AP waveform recorded from long photoperiod-entrained neurons at night, the time at which FDR current is increased in these cells. We also found that the AHP amplitude was increased and both AP and AHP durations were reduced in these neurons. The results indicate that photoperiodic encoding is accompanied by a selective modulation of the FDR current and consequently AP waveform, which may affect cellular communication.

### Most Single Cell Membrane Properties Do Not Contribute to the SCN Photoperiodic Phase Adjustment

It has been shown previously in a 12:12 photoperiod that the electrical activity, membrane potential, and input resistant are rhythmically controlled with higher values during the day compared with the night. Extracellular recordings of single- and multiunit activity revealed that individual unit activity patterns are not modified by photoperiod, while the waveform of collective electrical activity extends or compresses in long-day and short-day photoperiod, respectively (Vanderleest et al., 2007; Brown and Piggins, 2009). Consistent with previous research, our intracellular recording of single SCN neurons shows that the frequency of neuronal electrical activity remains unchanged in different photoperiods. Resting membrane potential is more depolarized during the day compared with the night and is important for regulation of the firing frequency as it increases the probability of triggering an action potential during the day (Schaap et al., 1999). The membrane potential in SCN neurons is regulated by a yet to be identified K^+^ current whose modulation is reflected by a decrease in input resistance during the night as compared with the day (De Jeu et al., 2002; Kuhlman and McMahon, 2004). We found that photoperiod did not affect the circadian modulation or amplitude of either resting membrane potential or membrane resistance. This implies that K^+^ conductances involved in regulation of resting membrane potential do not contribute to seasonal adaptation, albeit their role in daily rhythms.

### FDR Current and Action Potential Waveform Are Modulated by Photoperiod

In SCN neurons, the activity of FDR currents are under circadian control and contributes to the rhythm in electrical activity by enhancing repolarization of the AP and increasing firing rate during the day (Itri et al., 2005). Thus, modification in the amplitude of this current is expected to modify the electrical activity. However, in the present study, we detected elevated nighttime FDR current amplitude whereas the AP frequency was unchanged. Although the frequency does not change in response to the increased FDR current in long photoperiod at night, the AP waveform is considerably influenced by it. It is known that FDR current influences AHP shape and decreases the repolarization time of APs in the SCN and other brain regions (Martina et al., 1998; Porcello et al., 2002; Itri et al., 2005; Pedroarena, 2011). Increased FDR current at night in long-day photoperiod is therefore possibly causing the narrower APs with larger and faster AHP. Changes in the AP shape often alter the firing frequency. However, in long-day photoperiod, the membrane potential remained hyperpolarized at night, which may have prevented the modulation of firing frequency by the increased FDR current and transformed APs. Still, alteration of AP waveform by the FDR current could change transmitter release and influence the cellular communication (Ishikawa et al., 2003; Goldberg et al., 2005).

The modulation of FDR current by photoperiod raise the question of whether this current may be involved in encoding day length in SCN neuronal network. Intercellular signaling pathways using GABA and VIP are reported to contribute to the phase distribution of neuronal activity patterns within the SCN that underlies seasonal adaptation (Lucassen et al., 2012; Evans et al., 2013; Myung et al., 2015) and are therefore potential targets for FDR-mediated modulations of phase distribution.

### FDR Current May Contribute to Photoperiodic Regulation in the SCN

The results of the current study revealed that FDR current in the SCN may have other roles than modulating the firing rate. FDR channels deficient mice (lacking Kv3.1 and Kv3.2) show a disturbed behavioral phenotype with low amplitude and fragmented circadian patterns in locomotor activity as well as deficits in synchronizing to the environmental light cycle (Kudo et al., 2011). In the context of our data, it is of special interest that FDR current contributes to long-range synchronization between inhibitory interneurons of the neocortex (Harvey et al., 2012). Long-range cell-to-cell connections between ventrolateral and dorsomedial regions of the SCN may play a role in maintaining a narrow phase distribution within the SCN in short-day photoperiod as a simulation study suggested (Bodenstein et al., 2012). Moreover, long-range connections are important for synchronization of the neuronal activity and clock gene expression to photic information between dorsomedial and ventrolateral regions of the SCN (Leak et al., 1999; Welsh et al., 2010). The dorsomedial region of the SCN receives the light input indirectly through the ventrolateral region. Both VIP and gastrin-releasing peptide, are known to distribute photic information throughout the SCN network, have been shown to increase the FDR current in the dorsal SCN (Gamble et al., 2011; Kudo et al., 2013). Thus, the FDR current, by shaping the AP and possibly synaptic efficiency, may contribute to long-range communication within the SCN neuronal network. If so, the enhancement of the FDR current during the day in 12:12 or short-day photoperiods may be instrumental to determine the degree of synchronization. The reversal of circadian modulation of FDR in long-day photoperiod may lead to weakening of long-range functional connections and a wider distribution of phases. Therefore, a reversed rhythm in FDR amplitude may contribute to the plasticity of the SCN network in response to seasonal changes.

### FDR Current in Aging and Seasonal Adaptation

An increase in FDR current amplitude at night has also been found in SCN neurons from mice older than 24 months (Farajnia et al., 2012). There are noticeable resemblances between the aged phenotype and long photoperiod (Table 1), such as a short duration of behavioral activity, a low amplitude of multiunit electrical activity rhythm, an increased phase distribution of neuronal activity, and a reduction in phase shifting capacity (Rohling et al., 2006; Vanderleest et al., 2007; Biello, 2009; Vanderleest et al., 2009; Nakamura et al., 2011; Farajnia et al., 2012). The present study identified another similarity between the effects of long-day and aging on the SCN (Farajnia et al., 2012), which is an elevated FDR current at night. We propose that the reversion in FDR rhythm by long photoperiod is part of the physiological mechanism of day-length encoding. In contrast, the lack of circadian modulation of FDR in the old SCN may be a consequence of age-related functional decline as also seen for other membrane currents like the transient A-type K^+^ current (Farajnia et al., 2012) and large conductance Ca^2+^ activated K^+^ current (Farajnia et al., 2015). Given this data, it is possible that the age-related increase in FDR current during the night contributes to the desynchronization observed in the aged SCN network.

**Table 1.** Aging Resembles Long Photoperiod in Some Aspects but Not in Cellular Functions.

	Aged vs. young		Long vs. short days		References
Phase shifting capacity	Reduced		Reduced		Biello (2009), Vanderleest et al. (2007)
Electrical rhythm amplitude	Low		Low		Vanderleest et al. (2007), Farajnia et al. (2012), Nakamura et al. (2011)
Behavioral activity duration	Short		Short		Vanderleest et al. (2007), Farajnia et al. (2012)
Electrical activity pattern	Anti-phase		Broad		Vanderleest et al. (2007), Farajnia et al. (2012)
Membrane potential	–	↑	–	–	Farajnia et al. (2012), current study
Membrane resistance	–	↑	–	–	Farajnia et al. (2012), current study
Firing rate	↓	–	–	–	Vanderleest et al. (2007), Farajnia et al. (2012), Aujard et al. (2001), Watanabe et al. (1995), current study
I_A_ current	↓	–	–	–	Farajnia et al. (2012), current study
FDR current	–	↑	–	↑	Farajnia et al. (2012), current study
SDR current	–	–	–	–	Farajnia et al. (2012), current study
GABAergic current	↓	↓	↑	–	Farajnia et al. (2012), Nygard et al. (2005), Farajnia et al. (2014)
AP duration	–	↑	–	↓	Farajnia et al. (2015), current study
AHP amplitude	–	↓	–	↑	Farajnia et al. (2015), current study
AHP duration	–	–	–	↓	Farajnia et al. (2015), current study

*Note.* ↑ = increase; ↓ = decrease; – = no change in the mentioned property; FDR = fast delayed rectifier. Aged is compared with young and long day is compared with short-day photoperiod. When a cell is divided to white and gray background, it indicates the changes during the day and at night, respectively.

In aging, cellular deficiencies change the SCN output and generate a less effective signal. However, it seems that photoperiodic adaptation to long days implies a readjustment of cellular functions to control and support interneuronal communications. For instance, GABAergic signaling, which is important for interneuronal communication, is altered in long-day photoperiod as a consequence of molecular changes in the GABA equilibrium potential (Farajnia et al., 2014). In this study, we report a reversed circadian modulation of FDR current which may affect the long-range communication by reshaping the AP waveform.

In summary, we conclude that the circadian modulation of FDR current is affected by the seasonal changes in day length, which may contribute to phase distribution within the SCN during the long-day photoperiod. FDR currents were also shown to be crucial for the proper response of the central clock to photic information from the cyclic environment (Kudo et al., 2011). Both functions require long-range neuronal communication and synchronization within the SCN network which we suggest can be modulated by the FDR current. The better understanding of clock function on the level of ionic channels can thus lead to potential targets for intervention of circadian rhythms disturbances caused by aging or seasonal changes in day length like seen in seasonal affective disorders.
